# Molecular Modeling Study on the Allosteric Inhibition Mechanism of HIV-1 Integrase by LEDGF/p75 Binding Site Inhibitors

**DOI:** 10.1371/journal.pone.0090799

**Published:** 2014-03-05

**Authors:** Weiwei Xue, Huanxiang Liu, Xiaojun Yao

**Affiliations:** 1 State Key Laboratory of Applied Organic Chemistry, Department of Chemistry, Lanzhou University, Lanzhou, China; 2 School of Pharmacy, Lanzhou University, Lanzhou, China; 3 State Key Laboratory of Quality Research in Chinese Medicine, Macau Institute for Applied Research in Medicine and Health, Macau University of Science and Technology, Taipa, Macau, China; UMR-S665, INSERM, Université Paris Diderot, INTS, France

## Abstract

HIV-1 integrase (IN) is essential for the integration of viral DNA into the host genome and an attractive therapeutic target for developing antiretroviral inhibitors. LEDGINs are a class of allosteric inhibitors targeting LEDGF/p75 binding site of HIV-1 IN. Yet, the detailed binding mode and allosteric inhibition mechanism of LEDGINs to HIV-1 IN is only partially understood, which hinders the structure-based design of more potent anti-HIV agents. A molecular modeling study combining molecular docking, molecular dynamics simulation, and binding free energy calculation were performed to investigate the interaction details of HIV-1 IN catalytic core domain (CCD) with two recently discovered LEDGINs BI-1001 and CX14442, as well as the LEDGF/p75 protein. Simulation results demonstrated the hydrophobic domain of BI-1001 and CX14442 engages one subunit of HIV-1 IN CCD dimer through hydrophobic interactions, and the hydrophilic group forms hydrogen bonds with HIV-1 IN CCD residues from other subunit. CX14442 has a larger *tert*-butyl group than the methyl of BI-1001, and forms better interactions with the highly hydrophobic binding pocket of HIV-1 IN CCD dimer interface, which can explain the stronger affinity of CX14442 than BI-1001. Analysis of the binding mode of LEDGF/p75 with HIV-1 IN CCD reveals that the LEDGF/p75 integrase binding domain residues Ile365, Asp366, Phe406 and Val408 have significant contributions to the binding of the LEDGF/p75 to HIV1-IN. Remarkably, we found that binding of BI-1001 and CX14442 to HIV-1 IN CCD induced the structural rearrangements of the 140 s loop and oration displacements of the side chains of the three conserved catalytic residues Asp64, Asp116, and Glu152 located at the active site. These results we obtained will be valuable not only for understanding the allosteric inhibition mechanism of LEDGINs but also for the rational design of allosteric inhibitors of HIV-1 IN targeting LEDGF/p75 binding site.

## Introduction

Human immunodeficiency virus type 1 (HIV-1) is a retrovirus that causes acquired immunodeficiency syndrome (AIDS) [1], [2]. Integration is a crucial step in the HIV-1 life cycle mediated by the highly conserved and essential viral integrase (IN) protein. IN acts on the viral DNA attachment sites at the ends of the linear reverse transcript to effectively insert the reverse transcript into a host cell chromosome in a two-step reaction: 3′-processing and strand transfer [3]–[5]. Following 3′-processing, the IN protein removes two nucleotides from each 3′ end of the viral DNA, leaving recessed CA hydroxyl group at the 3′ end. After this cleavage, the IN protein remains bound to the viral DNA and joins the previously 3′ end to the 5′ end of strands of host cell chromosomal DNA.

Because of HIV-1 IN is essential for viral replication, it has become one of the most important therapeutic target. Many integrase strand transfer inhibitors (INSTIs) with different chemical scaffolds targeting the strand transfer reaction of HIV-1 IN, have been developed (e.g., raltegravir [6], elvitegravir [7], and dolutegravir [8]). Raltegravir is the first INSTIs approved by the U.S. Food and Drug Administration (FDA) in 2007 [9]. Elvitegravir, a second INSTIs, is recently approved by the FDA for the use in the treatment of HIV-1 infection in treatment-naïve adults [10]. FDA approved dolutegravir, a new drug to treat HIV-1 infection recently [11].

Although the great achievements in the development of the INSTIs class in recent years, the rapid emergence of viral strains that are highly cross-resistant to INSTIs has become a critical problem in INSTIs-based therapies [66]. Thus, new approaches to block the integration process are also in progress. For example, instead of targeting the strand transfer reaction catalytic activity of HIV-1 IN, several sites on HIV-1 IN have been identified as potential modulators of protein-protein interactions (PPIs) including the Lens Epithelium Derived Growth Factor (LEDGF)/p75 binding site [12]–[14]. LEDGF/p75 is a key cellular cofactor of HIV-1 IN that promotes viral integration by tethering the preintegration complex (PIC) to the host cell chromatin [15]–[17]. As the cofactor contributes to optimal viral replication, targeting LEDGF/p75-IN interactions has become a new potential therapeutic target for antiviral drug design [18]–[20].

**Figure 1:**
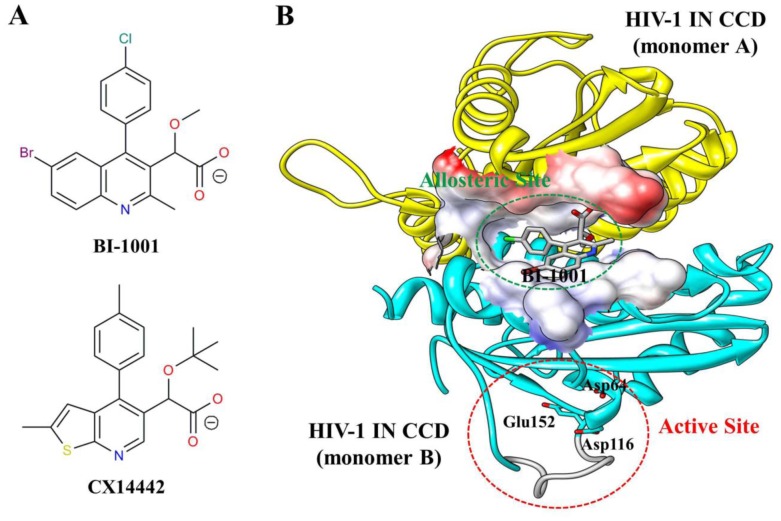
Structure of LEDGINs and HIV-1 IN CCD dimer. (A) Chemical structures of BI-1001 and CX14442. (B) Crystal structure of BI-1001 bound to the HIV-1 IN CCD dimer interface (PDB ID code 4DMN). The monomers are distinguished in yellow and cyan, and the BI-1001 is shown in gray stick model. The constructed missing loop (residues 141 to 151) is colored gray. The allosteric site at the HIV-1 IN CCD dimer interface is represented by surface. HIV-1 IN active site residues (Asp64, Asp116, and Glu152) are shown in cyan stick.

In recent years, several small molecule inhibitors of the LEDGF/p75 binding site of integrase (LEDGINs) that engage the LEDGF/p75 binding pocket of HIV-1 IN were discovered [21]–[24]. The LEDGF/p75 binding pocket locates at the catalytic core domain (CCD) dimer interface of HIV-1 IN and is distal from the active site (Figure 1). LEDGINs are found to inhibit LEDGF/p75-IN binding and IN catalytic activity *in vitro*, and HIV-1 replication in cell culture [21]–[24]. Most importantly, the developments of these allosteric HIV-1 IN inhibitors have opened a new route to overcome the cross-resistance problem of active site-based INSTIs [19]. However, except the recently reported CX14442 [21], the majority of discovered LEDGINs displayed low potency in cell culture [22]. To develop more potent inhibitors, the understanding about detailed molecular mechanism about LEDGF/p75 bound to the HIV-1 IN CCD dimer interface as well as the allosteric inhibition mechanism of LEDGINs on HIV-1 IN catalytic activity is very crucial.

Molecular modeling methods, such as molecular docking, molecular dynamics (MD) simulation and binding free energy calculation, have been proved to be very useful tools for protein-ligand and protein-protein interactions study [25]–[38]. In this work, using an combined computational approach, we explored the structural and energetic properties of the recently discovered two LEDGINs BI-1001 and CX14442, as well as the LEDGF/p75 bound to the HIV-1 IN CCD dimer interface. We expect that our computational results will provide useful information for understanding the allosteric inhibition mechanism of LEDGINs and further rational design of more potent allosteric HIV-1 IN inhibitors.

## Materials and Methods

### Construction of the Initial Structures

In order to study the allosteric inhibition mechanism of HIV-1 IN by LEDGINs, three systems were constructed and studied by using molecular modeling methods. They include HIV-1 IN CCD dimer in complex with LEDGINs BI-1001, CX14442, and host protein LEDGF/p75. The details about the complexes construction are described as follows. The initial structure for HIV-1 IN CCD in complex with BI-1001 was obtained from the Protein Data Bank (PDB ID code 4DMN [22]). First, the loop region encompassing residues 141–151 in HIV-1 IN CCD active site was predicted and refined with the program Prime [39]. Then, the transformation matrices method in VMD (v1.87) [40] was applied to build the HIV-1 IN CCD dimer. The initial structure of HIV-1 IN CCD dimer in complex with LEDGF/p75 was taken from the X-ray crystal structure with PDB ID code 2B4J [12]. Using the program Prime [39], the missing residues in chain A (187–194 and 187–194) and chain B (188–194) of 2B4J were constructed respectively using chain B and chain A in 2B4J as template. The structures were then processed by Protein Preparation Wizard in Schrödinger Suite 2009 before MD simulation.

The coordinates for the CX14442 bound HIV-1 IN CCD dimer complex were generated with the molecular docking method. The initial structure of CX14442 was constructed using Maestro [41] and was further processed by using LigPrep [42] based on MMFFs force field [43]. The protonation state of CX14442 was assigned using the program Epik [44] at a target pH value of 7.0±2.0. The structure of HIV-1 IN CCD dimer with BI-1001 was used in the molecular docking study. Before docking the inhibitor into the allosteric site, the receptor protein structure was prepared including adding hydrogen atoms, assigning partial charges using the OPLS-2005 force field [45] and assigning protonation states. The minimization was terminated when the root mean square deviation (RMSD) reached a maximum value of 0.30 Å. The grid box was defined by centering on the BI-1001 at HIV-1 IN CCD allosteric site. The docking of CX14442 into the prepared grids was carried out using program Glide [46] with the default parameter, for which standard precision (SP) mode was used. The conformation of CX14442 and HIV-1 IN CCD with best interaction was selected for the further MD simulation.

### Molecular Dynamics Simulations

All the treated structures summarized in Table 1 were then modeled by using the program LEaP embedded in AMBER10 [47] with the standard AMBERFF03 force field [48] used for the protein. These systems were neutralized and immersed into a rectangular periodic box of TIP3P [49] water molecules. Sufficient solvent was added to provide a minimum distance of 10 Å between any protein atom and the edge of the box. The force field parameters for BI-1001 and CX14442 were created with the use of Antechamber program from AMBER10 [47], using General Amber Force Field (GAFF) [50] and restrained electrostatic potential (RESP) [51]–[53] partial charges (Tables S1 and S2). Geometry optimization and the electrostatic potential calculations were performed at the HF/6-31G* level of Gaussian09 suite [54].

**Table 1:** Summary of the simulation systems.

systems	starting structure	water moleculars	total atoms	simulation time
BI-1001	X-ray structure (PDB ID code 4DMN)	9440	33134	200 ns
CX14442	CX-14442 docked into 4DMN	10682	36874	200 ns
LEDGF/p75	X-ray structure (PDB ID code 2B4J)	22160	73926	100 ns

MD simulations were performed using AMBER10 [47] with the AMBER force field. Initially, energy minimization was carried out for each solvated complex. Each system was minimized by two steps, applying harmonic restraints with a force constant of 500.0 kcal/(mol⋅Å^2^) to all protein atoms and allowing all atoms to move freely in turn. In each step, energy minimization was performed by the steepest descent method for the first 3000 steps and the conjugated gradient method for the subsequent 2000 steps. In energy minimization, the tolerance threshold is 1×10^−4^ kcal/(mol⋅Å), and the non-bonded cutoff is 12.0 Å. After minimization, all systems were heated up from 0 to 310.0 K over 100 ps in the *NVT* ensemble and equilibrating to adjust the solvent density under 1 atm pressure over 50 ps in the *NPT* ensemble simulation by restraining all atoms of the structures with a harmonic restraint weight of 10.0 kcal/(mol⋅Å^2^). Additional three MD equilibrations of 50 ps each were performed with the decreased restraints weight from 5.0, to 1.0, to 0.1 kcal/(mol⋅Å^2^), respectively. These were followed by the last MD equilibration step of 50 ps by releasing all the restraints. Afterward, production MD simulations were carried out without any restraint on these three systems in the *NPT* ensemble at a temperature of 310.0 K and a pressure of 1 atm. An integration time step of 2 fs was used and coordinate trajectory was recorded every 1 ps for all the equilibration and production runs. During the simulations, periodic boundary conditions were employed and all electrostatic interactions were calculated using the particle-mesh Ewald (PME) method [55] with a dielectric constant of unity. For all simulations, a 12.0 Å cutoff was used to calculate the direct space sum of PME, and bond lengths involving bonds to hydrogen atoms were constrained using the SHAKE algorithm [56].

### Thermodynamic Calculation

Ligand binding free energy was calculated using Molecular Mechanics/Poisson-Boltzmann Surface Area (MM/PBSA) method [57]. This method for computing free energy of our last 20 ns MD simulation of equilibrated trajectories requires removal of solvent waters and counter ions. We collected 1000 snapshots for the complex, receptor, and ligand respectively from MD trajectory, equally spaced at 20 ps intervals, and the binding free energy was calculated according to the equation:
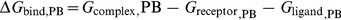
(1)where *G*_complex,PB_, *G*_receptor,PB_, and *G*_ligand,PB_ are the free energy of complex, receptor and ligand molecules, respectively. The free energy (*G*_bind,PB_) was calculated based on an average over the extracted snapshots from a single-trajectory MD simulation. Each state is estimated from the molecular mechanics energy *E*_gas_, the solvation free energy *G*_sol,PB_, and the solute entropy *S* as follows.



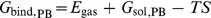
(2)

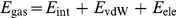
(3)




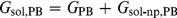
(4)




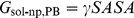
(5)where *E*_gas_ is the gas-phase energy; *E*_int_ is the internal energy; *E*_ele_ and *E*_vdW_ are the Coulomb and van der Waals energies, respectively. *E*_gas_ was calculated using the Amberff03 force field. *G*_sol,PB_ is the solvation free energy and can be decomposed into polar and nonpolar contributions. *G*_PB_ is the polar solvation contribution calculated by solving the PB equation [58]. Dielectric constants for solute and solvent were set to 1 and 80, respectively [58]. *G*_sol-np,PB_ is the nonpolar solvation contribution and was estimated by the SASA determined using a water probe radius of 1.4 Å. The surface tension constant *γ* was set to 0.0072 kcal/(mol/Å^2^) [59]. *T* and *S* are the temperature and the total solute entropy, respectively. Vibrational entropy contributions can be estimated by classical statistical thermodynamics, using normal mode analysis [60]. Normal mode calculations for the complex, receptor, and ligand and average the results were carried out with the NMODE module in AMBER10 [47] to find the entropic contributions. Due to the high computational cost in the entropy calculation, 20 snapshots for the complex, receptor, and ligand respectively were extracted from the last equilibrated 20 ns of the molecular dynamics simulations with 1000 ps time intervals and each snapshot was fully minimized with a distance dependent dielectric function 4R_ij_ (the distance between two atoms) until the root mean square of the elements of the gradient vector was less than 1×10^−4^ kcal/(mol⋅Å).

### Free Energy Decomposition Analysis

In order to investigate the contribution of each residue to the binding affinity, which is valuable to describe the binding mode of LEDGINs and LEDGF/p75 to HIV-1 IN CCD dimer, per-residue free energy decomposition analysis implemented in MM/GBSA module was performed by:
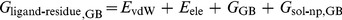
(6)where *E*_vdW_ and *E*_ele_ are non-bonded van der Waals interactions and electrostatic interactions were computed using the SANDER program in AMBER10. *G*_GB_ and *G*_sol-np,GB_ are the polar and nonpolar contributions to the inhibitor-residue interaction. The polar solvation contribution (*G*_GB_) was calculated by using the generalized Born (GB) model [61], and the parameters for the GB calculation were developed by Onufriev *et al.* (GB^OBC^, igb = 2). The nonpolar contribution of desolvation (*G*_sol-np,GB_) was computed based on SASA.

## Results and Discussion

### The Initial Structures of the Constructed Complexes

In the co-crystal structure of HIV-1 IN CCD with BI-1001 (PDB ID code 4DMN [22]), the residues from 141 to 151 in the active site were not solved [22]. Therefore, the missing 141 to 151 residues in 4DMN active site were directly predicted using the program Prime [39], and Figure 1B illustrates the model that has been constructed.

**Figure 2:**
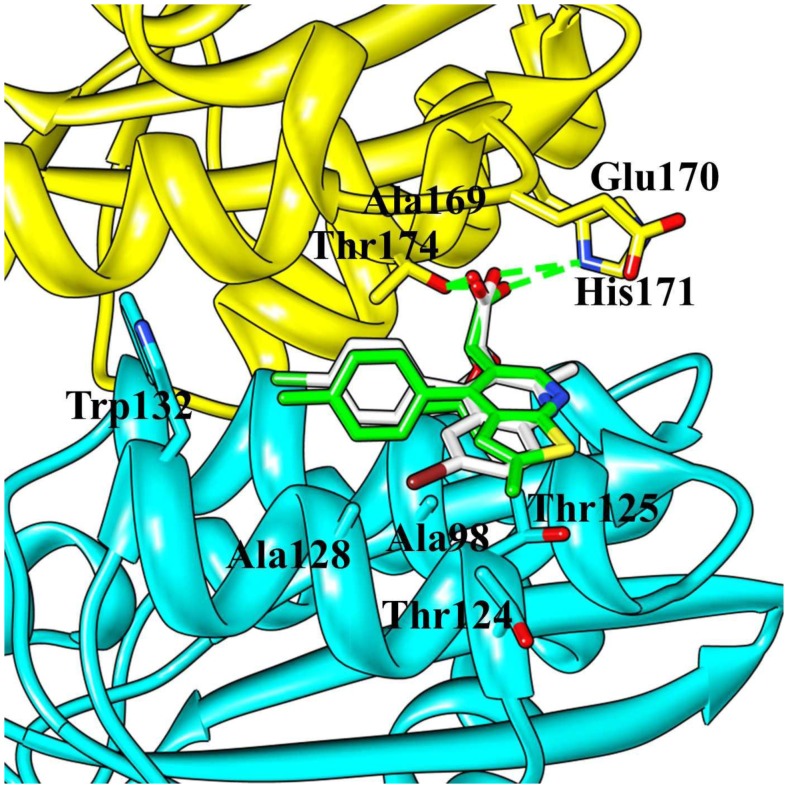
Comparison of the computational docking of CX14442 in the HIV-1 IN CCD dimer versus the reported crystal structure of BI-1001 bound in HIV-1 IN CCD dimer (PDB ID code 4DMN). The protein is shown in the cartoon representation; the two monomers are colored yellow and cyan, respectively. The LEDGINs are represented in gray stick. Hydrogen bond interactions are denoted by dotted green lines.

CX14442, the derivative of BI-1001, is a more potent inhibitor of HIV-1 IN that directly acting at the allosteric site [21]. Unfortunately, the crystal structure of HIV-1 IN CCD bound to the CX14442 has not been determined so far. Herein, on the basis of our modified co-crystal structure 4DMN, the interaction mode between the HIV-1 IN CCD allosteric site and the CX14442 was obtained by using molecular docking approach. The accuracy of the docking protocol has been checked by redocking the ligand BI-1001 into the crystal structure and the RMSD of the atomic positions between the ligand and the docked pose is 0.47 Å. Figure 2 illustrated the structures of BI-1001 and CX14442 binding at the allosteric site. As seen in Figure 2, CX14442 having favorable hydrophobic interactions with Ala98, Thr124, Thr125 and Trp132. Additionally, we found that CX14442 forms two important hydrogen bond interactions with His171 and Thr174. However, in comparison to BI-1001, the predicted binding pose of CX14442 exhibits better interaction with the allosteric site of HIV-1 IN CCD.

In addition, the reported experimental study revealed that the conformational flexibility of HIV-1 IN CCD active site loop is important for the catalytic step of inserting the viral DNA into the host chromosomal DNA [64]. By comparing the active site loop of 2B4J and our modified co-crystal structure 4DMN, we found that the loop conformational changes occurred (Figure S1). This observation may explain the *in vitro* assay results that binding of BI-1001 at the allosteric site can affect the catalytic activity of HIV-1 IN [22]. Further MD simulations and binding free energies calculations were necessary to obtain the detailed interaction mode and relevant conformation change during the protein-ligand recognition process.

### Molecular Dynamics Simulations and the Stability of the Simulation Systems

**Figure 3:**
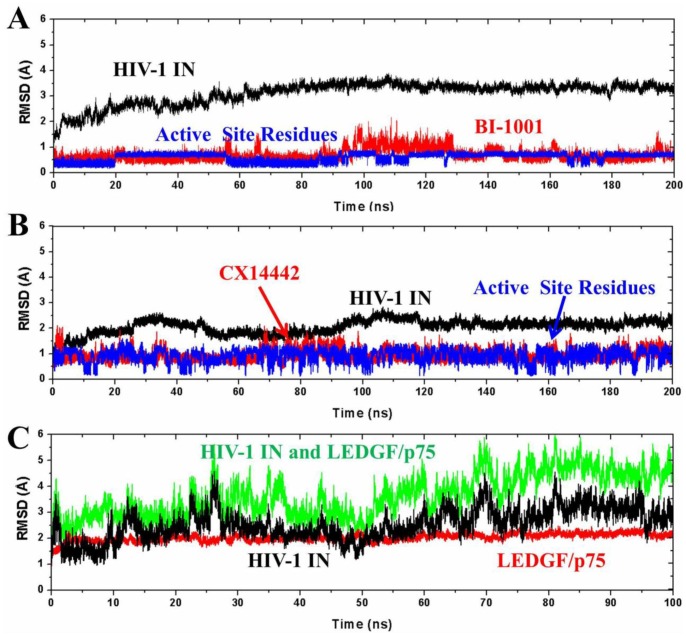
The monitored RMSD of the backbone atoms of protein (black), backbone atoms of binding pocket residues around 5 Å of ligand (blue), and the heavy atoms in the ligand (red) for: (A) BI-1001 and (B) CX14442 bound HIV-1 IN complexes with respect to the initial structures as a function of time. (C) The monitored RMSD of the backbone atoms of HIV-1 IN and LEDGF/p75 (black), backbone atoms of HIV-1 IN (blue), and backbone atoms of LEDGF/p75 (red) for LEDGF/p75 bound HIV-1 IN complex with respect to the initial structures as a function of time.

Based on the designed three models, a total of 500 ns MD simulations were carried out to investigate the protein-ligand interaction efficacy and the role of the binding inhibitors BI-1001 and CX14442 to the active site conformational changes. The simulations were monitored by determining the root-mean-square deviation (RMSD) of the backbone atoms for each protein relative to the initial coordinates of the simulated systems (Figure 3). Herein, analysis of the RMSD for the active site residues (around 5 Å of ligand) backbone atoms and ligand heavy atoms are also illustrated. As seen from Figure 3, the protein backbone atoms RMSD of BI-1001, CX14442, and LEDGF/p75 bound HIV-1 IN CCD in the simulation fluctuates around 3.3 Å, 2.2 Å, and 2.1 Å after 100 ns, 120 ns, and 70 ns, respectively. However, the behavior of the RMSD shown in Figure 3B indicated that CX14442 sometimes follows the RMSD of the binding site residues. This is because the initial structure of CX14442 bound to HIV-1 IN was obtained by using docking method. Compared to the X-ray structure of the BI-1001 in complex with HIV-1 IN CCD, the CX1442 bound structure is not enough reasonable. Therefore, CX14442 searches a more reasonable conformation to accommodate the binding site during the MD simulation. Figure 3C gives the monitored RMSD of the backbone atoms of HIV-1 IN CCD and LEDGF/p75, backbone atoms of HIV-1 IN CCD, and backbone atoms of LEDGF/p75 for LEDGF/p75 bound HIV-1 IN complex with respect to the initial structure as a function of time. The large value of the RMSD (red) shown in Figure 3C is contributed by both HIV-1 IN and LEDGF/p75. Overall, all the simulated systems proved to be stable after MD simulations, which implies that the protein-ligand/protein complexes have reached a stable equilibrated conformational state. In following contents, we will discuss in detail about binding modes of the MD-simulated structures of protein binding with LEDGINs and LEDGF/p75, and then discuss the MM/PBSA calculation of binding free energy. After this, a detailed discussion of the conformational changes of HIV-1 IN active site by LEDGINs binding is presented.

### Structures and Energies of LEDGINs and LEDGF/p75 Binding to the HIV-1 IN

#### Essential residues of HIV-1 IN contribute to binding of the LEDGINs

**Figure 4:**
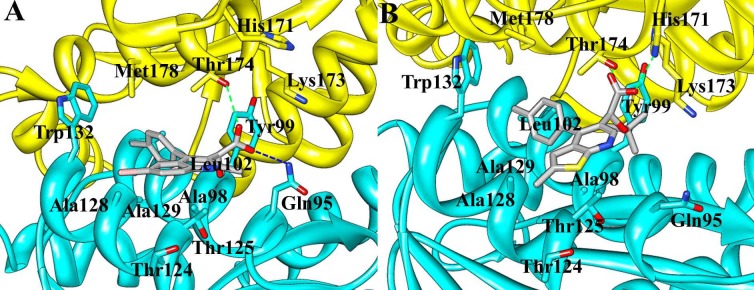
Structural models of (A) BI-1001, (B) CX14442 in complex with HIV-1 IN CCD dimer. The averaged structures extracted from the MD trajectories were used. The proteins are shown in cartoon representation with two monomers in yellow and cyan. The BI-1001 and CX14442 are shown in gray stick model. Hydrogen bond interactions are denoted by dotted green or blue lines.

**Figure 5:**
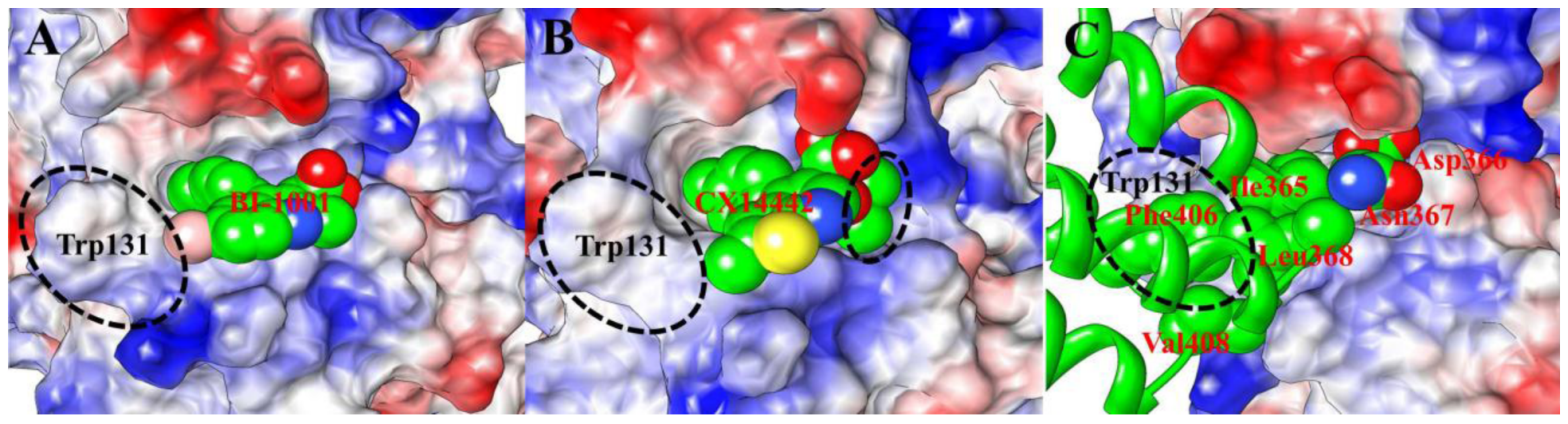
Electrostatic potential surface of the allosteric binding pocket of HIV-1 IN CCD dimer in interaction with (A) BI-1001, (B) CX14442, and (C) LEDGF/p75. The positive charges are displayed in blue, negative charges are displayed in red, and neutral residues are displayed in white. Color intensity is proportional to the charge value. The BI-1001, CX14442 and side chain of the LEDGF/p75 key residues, whose carbon atoms are shown as green spheres and labeled as red. The residue Trp131 from monomer A of HIV-1 CCD dimer is also labeled (black).

Understanding protein-ligand binding processes is undoubtedly of critical importance in structure-based drug design. D77, as the first small molecule targeting the LEDGF/p75 binding site in CCD dimer interface, provided useful information for the discovery and development of new anti-HIV agents [67]. From molecular docking with site-directed mutagenesis analysis and surface plasmon resonance (SPR) binding assays, the key residues Gln95, Thr125, Trp131, and Thr174 A were proved to play important roles for the binding of D77 to HIV-1 IN CCD [67]. Figure 4 and Figure 5A, 5B gives the MD-averaged structures of the LEDGINs BI-1001 and CX14442 bind to the HIV-1 IN CCD dimer interface, and with the atomic coordinates provided in the Supporting Information (PDB S1, S2). The carboxyl groups of BI-1001 and CX14442 form hydrogen bond with the side chain oxygen of Thr174, and the hydrophobic or aromatic moiety of BI-1001 and CX14442 primarily engages another HIV-1 IN CCD monomer through hydrophobic interactions.

**Figure 6:**
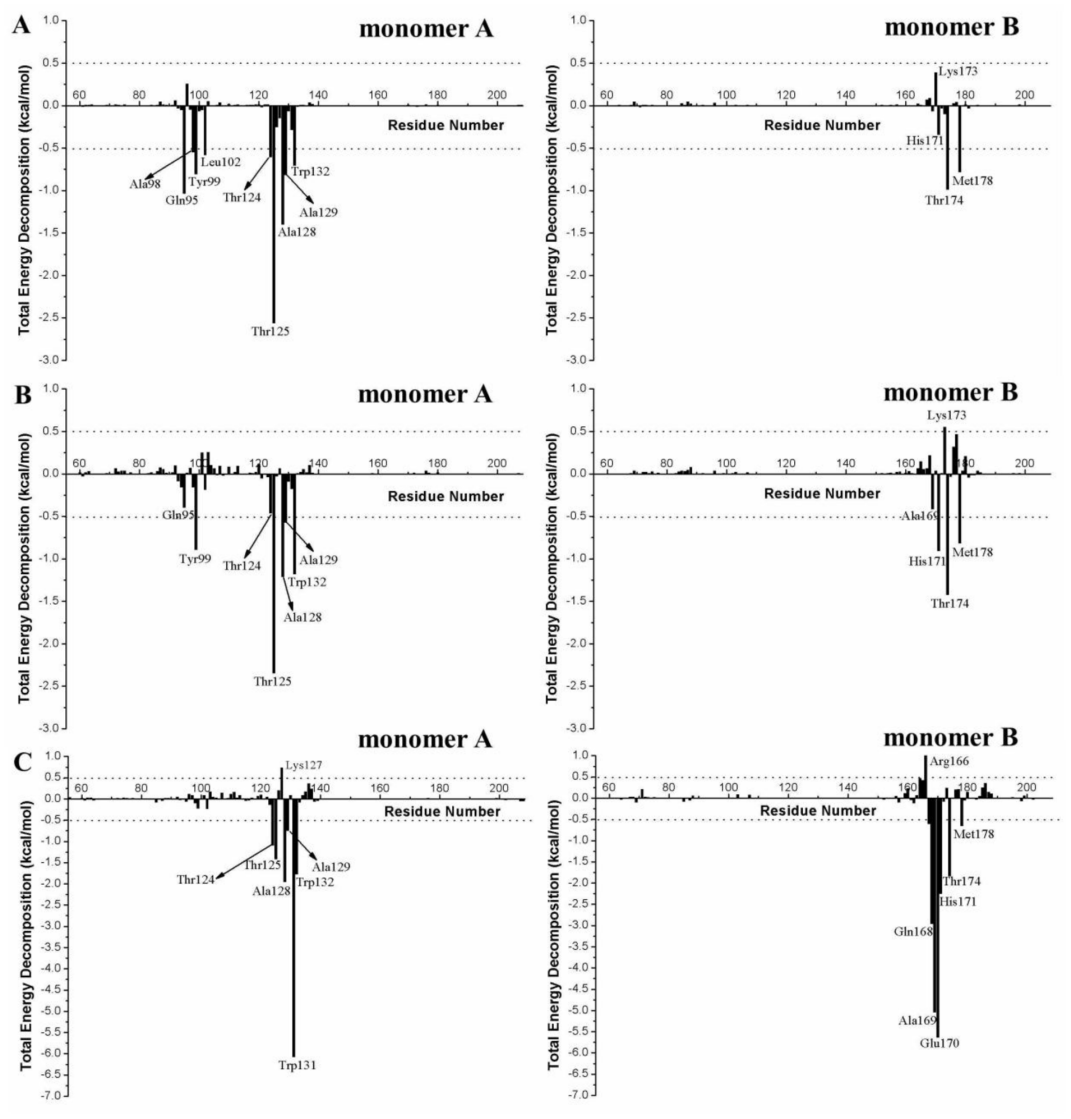
Per-residue interaction spectrum of the residues of HIV-1 IN CCD with (A) BI-1001, (B) CX14442, and (C) LEDGF/p75 in complex with the HIV-1 IN CCD dimer from MM/GBSA free energy decomposition analysis.

In order to identify the residues responsible for the difference in potency of BI-1001 and CX14442 against HIV-1 IN, the contribution of each residue to the binding free energies was calculated for both BI-1001 and CX14442 bound complexes. As can be seen in Figure 6A and 6B, the key residues contribution to the total binding free energies of the studied systems including the residues Gln95, Ala98, Tyr99, Leu102, Thr124, Thr125, Ala128, Ala129, and Trp132 from one subunit of HIV-1 IN CCD dimer and Ala169, His171, Thr174, and Met178 from the other subunit. Among them, although residues Gln95, Ala98, Tyr99, Leu102, and Ala129 bind stronger to BI-1001, it can be found that the residues Tyr99, Trp132, Ala169, His171, and Thr174 are more favorable to CX14442 binding.

In the MD-simulated structures, carboxyl group of BI-1001 forms hydrogen bond interactions with the side chain of residues Gln95 and Tyr174 (Figure 4A) from one monomer of HIV-1 IN CCD. Comparisons of the co-crystal structure (Figure 2B), BI-1001 lost the hydrogen bond with His171 from its carboxyl acid oxygen atom. However, it is possible to maintain this important hydrogen bond interaction for CX14442 (Figure 4B). This difference may be from the *ter*-butyl of CX14442 which make stronger interactions with the side chain of Tyr99 (Figure 6B), assisting CX14442 to adopt a more rational orientation that enables the interactions between the oxygen atom of the carboxyl group and protein His171 (Figure 4B).

Additionally, as shown in Figure 4, the hydrophobic or aromatic moiety of the BI-1001 and CX14442 primarily engages another HIV-1 IN CCD monomer through hydrophobic interactions. Nonetheless, it is clear that BI-1001 cannot be positioned to enable the chlorophenyl moieties to have strong interact with the aromatic side chain of the residue Trp132 (Figure 4A and Figure 6A), whereas CX14442 forms better interactions with the hydrophobic environment (especially the residue Trp132) of HIV-1 IN (Figure 4B and Figure 6B).

**Figure 7:**
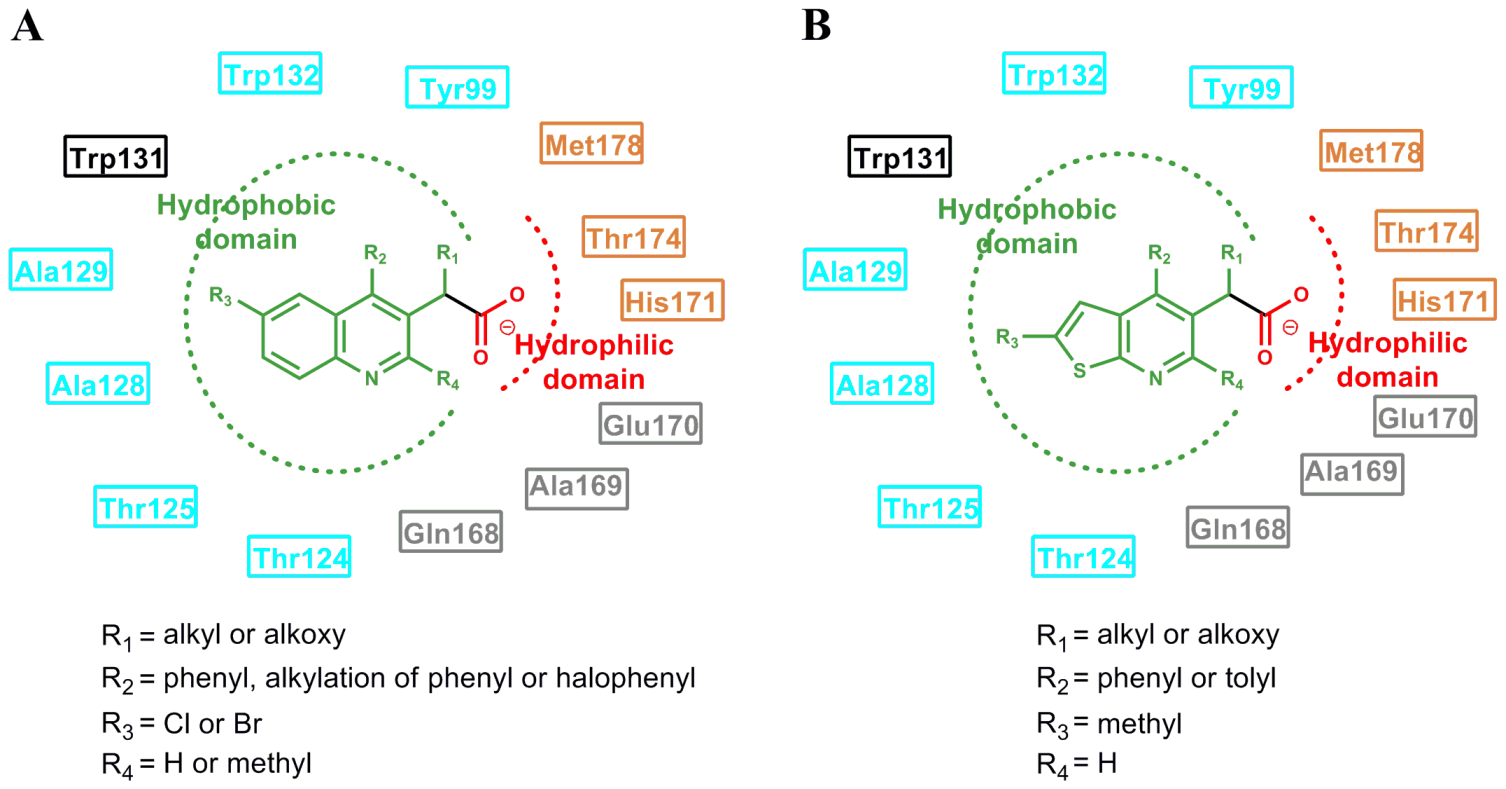
The generated pharmacophore for HIV-1 IN LEDGINs based on the receptor-ligand interactions. The model consists of hydrophobic and hydrophilic features on LEDGINs as well as the key residue in HIV-1 IN allosteric site. The hydrophobic and hydrophilic domains are shown in green and red, respectively. The residues that participated in the interaction between LEDGINs and HIV-1 IN CCD are labeled in cyan and orange, while the potential residues used for further extension LEDGINs design are labeled in black and gray.

From the above discussion, a general pharmacophore model based on the protein-ligand interactions was generated and shown in Figure 7. The illustration of BI-1001 and CX14442 fit to the pharmacophore is shown in Figure 7A and 7B, respectively. The structural difference in these two compounds is the substituent group R_2_ as shown in the pharmacophore model. The higher efficiency of the latter is mainly due to the fact that the *tert*-butyl ether could assistant CX14442 perfectly accommodate the allosteric site (Figure 4B and 5B). This is in agreement with the fact that the increase of the hydrophobic interactions will improve the activity [21]. Meanwhile, the steric effects of the bulkier group play an important role in controlling the orientation of carboxyl acid oxygen to keep hydrogen bond interactions with His171 (Figure 4B). Therefore, we expected that the understanding of the detailed mode of LEDGINs action might provide useful information for the rational structure-based drug design of more potent anti-HIV agents.

#### Recognition mechanism of HIV-1 IN by LEDGF/p75

**Figure 8:**
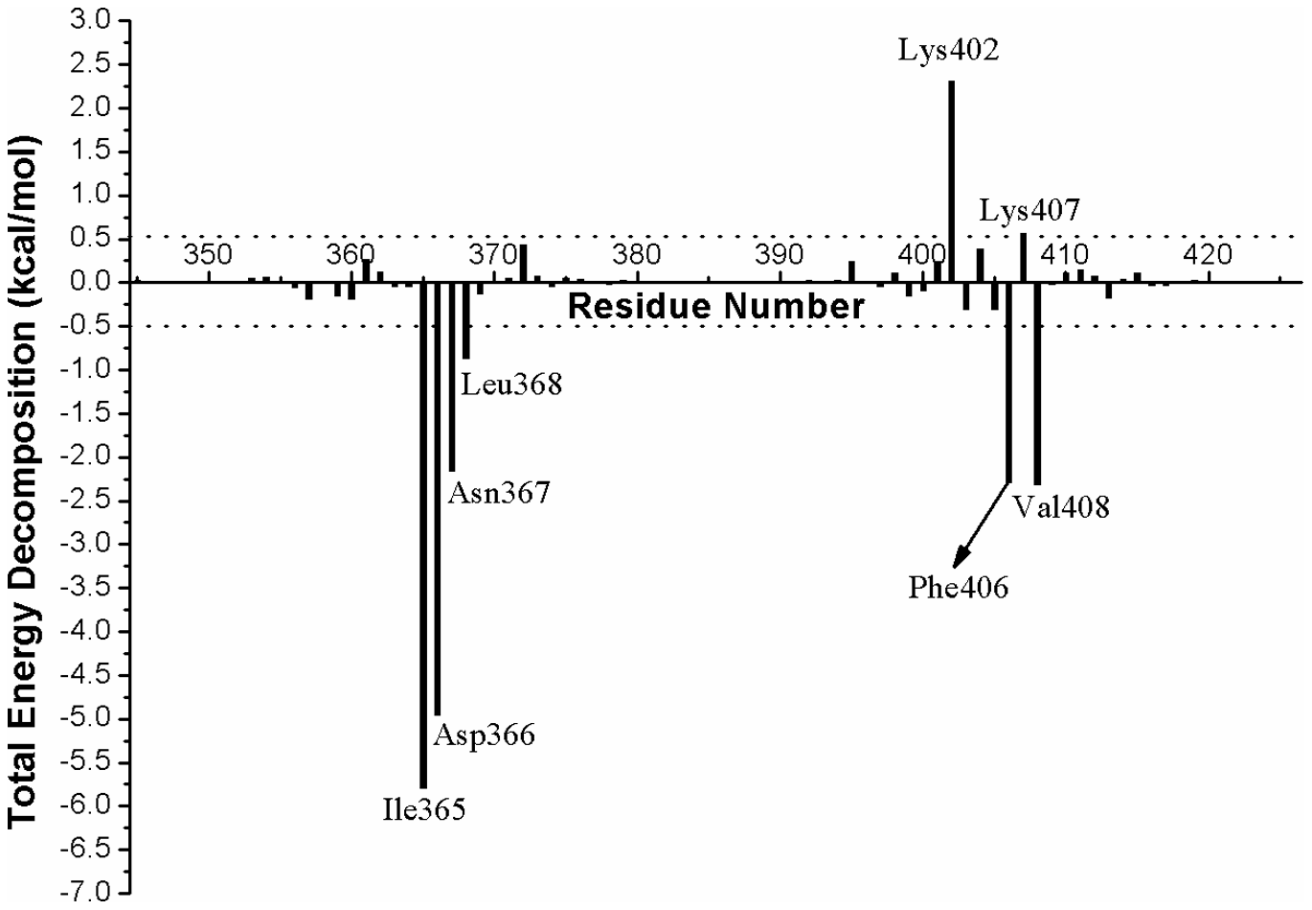
The LEDGF/p75 protein residues contribution to the total binding free energy of the LEDGF/p75 bound HIV-1 IN CCD complex.

**Figure 9:**
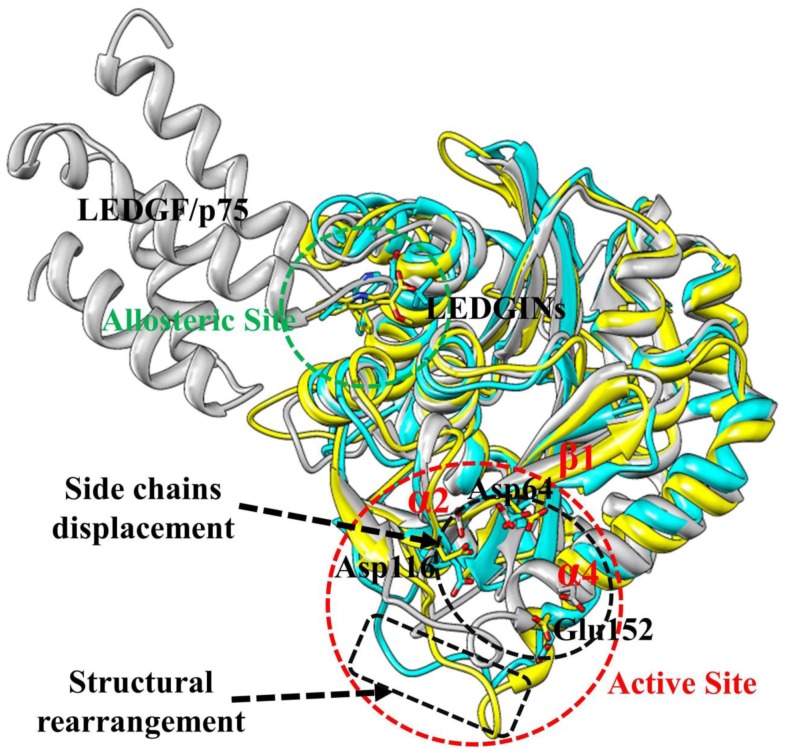
Structural model of LEDGF/p75 in complex with HIV-1 IN CCD dimer. The averaged structure extracted from the MD trajectory was used. The protein is shown in cartoon representation with two monomers in yellow and cyan. The side chains of the LEDGF/p75 amino acids are shown as gray sticks. Hydrogen bond interactions are denoted by dotted green lines.

HIV-1 IN leans heavily on interactions with LEDGF/p75 during the crucial step of the viral life cycle [15]–[17]. Figure 5C show the molecular surface representation of the average binding pocket structure of the LEDGF/p75 bound to the HIV-1 IN CCD. According to the results of the free energy decomposition analysis, the residues Thr124, Thr125, Ala128, Ala129, Trp131, and Trp132 from one subunit of HIV-1 IN CCD dimer and Gln168, Ala169, Glu170, His171, Thr174, and Met178 from the other monomer (Figure 6C) interact with the residues Ile365, Asp366, Asn367, Leu368, Phe406, and Val408 of the LEDGF/p75 integrase binding domain (IBD) (Figure 8). Meanwhile, this detailed interaction mode was shown in Figure 9, and with the atomic coordinates provided in the Supporting Information (PDB S3). As can be seen in Figure 9, only one hydrogen bond interaction was identified between residue Asp366 of LEDGF/p75 and residue Thr174 of HIV-1 IN. However, it should be noted that the identified hot spots residues based on the simulated structure is consistent with the previously reported results [62].

In Figure 8, it reveals that the LEDGF/p75 integrase binding domain residues Ile365, Asp366, Phe406 and Val408 have significant contributions to the binding free energies of the LEDGF/p75-IN complex. The binding mode of the LEDGF/p75 to HIV-1 IN shown in Figure 5C demonstrates that the Ile365, Asp366, Phe406 and Val408 fits well at the defined binding cleft, particular the strong interactions between Phe406 and Trp131 (Figure 6C).

In recent years, by mimicking the binding of the LEDGF/p75 IBD residues Ile365 and Asp366 in the LEDGF/p75 binding site, a class of 2-(quinolin-3-yl) and 2-(thieno[2,3-b]pyridin-3-yl) acetic acid derivatives including the studied BI-1001 and CX14442 was identified as HIV-1 IN allosteric inhibitors [19], [21]–[23]. Thus, we here compared the binding mode of BI-1001, CX14442, and LEDGF/p75 with HIV-1 IN CCD. It is shown that BI-1001 and CX14442 only occupy the binding site of residues Ile365 and Asp366 in LEDGF/p75 IBD loop (Figure 5). However, our calculation results proved that the residue Phe406 and Val408 in the LEDGF/p75 IBD loop, as equally important with Ile365 and Asp366, were critical to the binding of LEDGF/p75 to the HIV-1 IN CCD. Therefore, to design of novel and more potent LEDGINs, a further extension of the strategy is the stretch of the Phe406 and Val408 binding sites of LEDGF/p75 IBD. Such a concept implies that the future designed inhibitors can interact not only with the certain residues labeled in orange and cyan shown in the pharmacophore model, but also with the potential residues which labeled in black and gray (Figure 7). However, we suggest that the strength of binding and the inhibition should be designed making a compromise between the hydrophobic interactions mostly on the border of the site and the polar interactions.

#### Binding free energies analysis

We performed MM/PBSA calculations to get quantitative estimation for the binding free energies and their components of HIV-1 IN in complexed with BI-1001, CX14442, and LEDGF/p75. The results were collected in Table 2. The predicted MM/PBSA binding free energies for HIV-1 IN CCD with BI-1001, CX14442, and LEDGF/p75 are −14.96 kcal/mol, −18.41 kcal/mol, and −40.42 kcal/mol, respectively, which is in good agreement to the order of the experimental activities [21], [22], [60]. It is reasonable that the predicted binding free energies can explain the fact that CX14442 has stronger binding ability than BI-1001. The difference in total nonpolar interaction energies (ΔE_vdW_+ΔG_SA,PB_) contributions in the CX14442 complex (−52.75 kcal/mol) compared to the BI-1001 complex (−49.24 kcal/mol) seems to be the main source for the stronger binding ability of CX14442 than BI-1001.

**Table 2:** The free energies calculated by the MM/PBSA methods for binding of BI-1001, CX14442, and LEDGF/p75 to the HIV-1 IN.

Systems	Contributation^a^								Δ*G*_bind,PB_ e	IC_50_ (µM) [21], [22], [65]
	Δ*E*_vdW_	Δ*G*_SA,PB_	Δ*E*_ele_	Δ*G*_PB_	Δ*E*_gas_ b	Δ*G*_sol,PB_ c	Δ*E*_bind,PB_ d	−*T*Δ*S*		
BI-1001	−27.90±0.08	−21.34±0.06	−22.30±0.58	36.56±0.54	−50.20±0.61	15.22±0.52	−34.98±0.16	20.02±1.28	−14.96	1.0±0.1
CX14442	−28.58±0.09	−24.17±0.07	−15.18±0.68	32.10±0.66	−43.76±0.71	7.93±0.54	−35.83±0.28	17.42±0.96	−18.41	0.046±0.012
LEDGF/p75	−53.29±1.04	−37.86±0.75	97.93±2.23	−70.12±2.64	44.64±3.15	−107.98±1.98	−63.34±1.29	22.92±4.62	−40.42	0.0043±0.0008

a All energies are in kcal/mol, with corresponding standard errors of the mean.

b Δ*E*_gas_ = Δ*E*_vdW_+Δ*G*_SA,PB_.

c Δ*G*_sol,PB_ = Δ*G*_SA,PB_+Δ*G*_PB_.

d Δ*E*_bind,PB_ = Δ*E*_gas_+Δ*G*_sol,PB_.

e Δ*G*_bind,PB_ = Δ*E*_bind,PB_−*T*Δ*S*.

Comparison of the binding free energy components listed in Table 2 demonstrates that van der Waals (Δ*E*_vdW_) and electrostatic terms (Δ*E*_ele_) in the gas phase provide the major favorable contributions to the inhibitor binding. In contrast, the total solvation energies (Δ*G*_sol,PB_ = Δ*G*_SA,PB_+Δ*G*_PB_), particularly the polar solvation energies (Δ*G*_PB_), have unfavorable contributions to BI-1001 and CX14442 binding energies.

However, it can be observed that the van der Waals (Δ*E*_vdW_) energy, polar (Δ*G*_PB_), and nopolar (Δ*G*_SA,PB_) solvation energies are favorable for the LEDGF/p75 binding, whereas the electrostatic energy (Δ*E*_ele_) is unfavorable for binding. Unlike the BI-1001 and CX14442 bound complexes, the favourable contribution of the polar solvation free energy indicated that thermodynamic forces leading to the presence of buried water molecules creating a network that bridge the LEDGF/p75 to the HIV-1 IN CCD. Meanwhile, this is consistent well with the water molecules detectable in the allosteric site of the crystallographic structure 2B4J [12], which further suggest that the complex formation is associated with solvation.

In addition, it is known that formation of macromolecular complex is opposed by a loss in configurational entropy (−*T*Δ*S*) of the binding partners. Herein, we estimated the corresponding entropy contributions upon binding of BI-1001, CX14442 and LEDGF/p75 for HIV-1 IN CCD which are ranging from 17.42 kcal/mol to 22.92 kcal/mol (Table 2). As shown in Table 2, the incorporation of an entropic term would enable us to accurately predict the final free energy binding.

### Conformational Changes of HIV-1 IN Active Site by LEDGINs Binding

**Figure 10:**
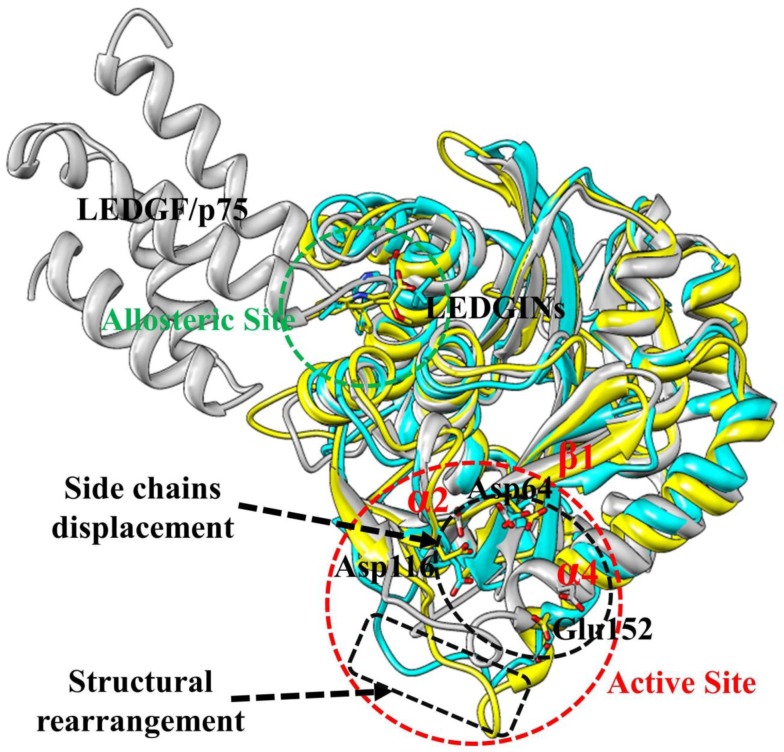
The aligned representative conformations of BI-1001, CX14442, and LEDGF/p75 bound HIV-1 IN CCD dimer models. The averaged structures extracted from the MD trajectories were used. The BI-1001, CX14442, and LEDGF/p75 bound form are shown in yellow, cyan and gray, respectively. HIV-1 IN active site residues (Asp64, Asp116, and Glu152) are shown in stick. The LEDGINs and LEDGF/p75 are represented in stick and carton, respectively.

**Figure 11:**
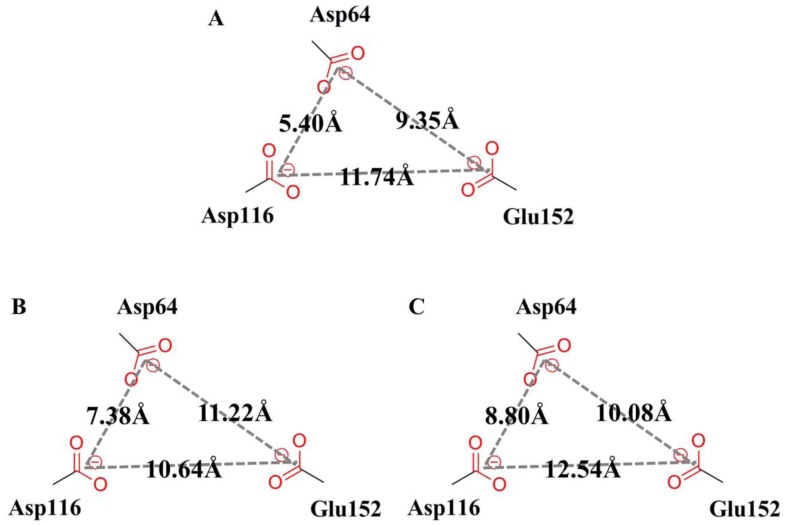
Scheme of the active site DDE motif (Asp64, Asp116, and Glu152) models for (A) LEDGF/p75, (B) BI-1001 and (C) CX14442 bound HIV-1 IN complexes. The measured distances between the centroid of the side chains of the three conserved catalytic residues were labeled in each model.

It is reported that the LEDGINs direct binding to the LEDGF/p75-binding pocket and these compounds function as allosteric inhibitors of HIV-1 IN activity [19]–[23]. Compared with the LDEGF/p75 bound structure, the active site of HIV-1 IN CCD appears to have two main conformational changes caused by the allosteric inhibition of BI-1001 and CX14442 (Figure 10). First, the 140 s loop (residue 141 to 149 in HIV-1 IN CCD) of BI-1001 and CX14442 bound systems undergo significant structural rearrangements inside of the active site of the enzyme. Second, in both BI-1001 and CX14442 bound complexes, displacement of the side chains of the conserved DDE motif (Asp64, Asp116, and Glu152) are observed (Figure 10). In order to monitor this displacement, we calculated distances between the centroid of the side chains of these three conserved catalytic residues (Figure 11). In the LEDGF/p75 bound HIV-1 IN complex (Figure 11A), the measured distance between the side chains of Asp64, Asp116, and Glu152 are 5.40 Å (Asp64···Asp116), 9.35 Å (Asp64···Glu152), and 11.74 Å (Asp116···Glu152), respectively. Compared with Figure 11A, there are great changes of the distance between Asp64 and Asp116, Asp64 and Glu152, and Asp116 and Glu152 in the BI-1001 and CX14442 bound HIV-1 IN complexes (Figure 11B and 11C).

On the basis of the experimental information, the conserved residues Asp64, Asp116, and Glu152 in the active site play an important role in 3′-processing, strand transfer, and disintegration activities of HIV-1 IN [63], and the conformation of the flexible 140 s loop at the active site is important for a post-binding catalytic step of HIV-1 replication [64]. Therefore, the proposed atomistic-level model of the allosteric inhibition mechanism of LEDGINs to the HIV-1 IN CCD active site shown in Figure 10 can help to understand the experimental results that LEDGINs engage the LEDGF/p75 binding site impair the inherent HIV-1 IN catalytic activity [19], [20], [22], [23].

## Conclusions

HIV-1 IN is a clinically validated target for designing novel antiviral therapies. LEDGINs are allosteric inhibitors that target the LEDGF/p75 binding site and interfere indirectly with the HIV-1 IN catalytic activity. However, it remains a challenge to develop more potent allosteric inhibitors. In the present study, molecular docking, MD simulations, binding free energies calculations and per-residue binding free energy decomposition were used to investigate the interactions of LEDGINs BI-1001, CX14442, and the LEDGF/p75 to HIV-1 IN CCD dimer interface. MD simulations combined with binding free energies calculations highlight the stronger binding abilities of CX14442 compared to BI-1001. Considering that the affinity of the inhibitors is determined by the hydrophobic environment in the allosteric site of the HIV-1 IN CCD dimer interface, and CX14442 has a greater *tert*-butyl group than BI-1001, lending the former a better affinity for the highly neutral binding pocket. In addition, by analyzing the generated pharmacophore model and the energetic decomposition results, it is able to provide some clue for the future rational drug design of more potent LEDGINs. Finally, our MD simulations results strongly suggested that structural rearrangements of the 140 s loop residues and the orientation changes of the side chains of the three conserved catalytic residues Asp64, Asp116, and Glu152 occur in HIV-1 IN CCD active site may be associated to LEDGINs binding to the allosteric site. In conclusion, the detailed understanding of the interaction mechanism of LEDGINs and the effect of binding upon active site conformation changes could aid the development of novel inhibitors and help explain the phenomenon observed by experiment.

## Supporting Information

Figure S1 **Structural models of BI-1001 and LEDGF/p75-bound HIV-1 IN CCD dimer complexes.** (A) The modified crystal structures of BI-1001 in complex with HIV-1 IN CCD (PDB ID code 4DMN). (B) The crystal structures of LEDGF/p75 in complex with HIV-1 IN CCD (PDB ID code 2B4J). The protein is shown in the cartoon representation; the two monomers are colored yellow and cyan, respectively. The flexible 140 s loop (residues 140–149) is colored gray. HIV-1 IN active site residues (Asp64, Asp116, and Glu152) are shown in cyan stick. The LEDGINs and LEDGF/p75 are represented in gray stick and carton, respectively.
(TIF)

Table S1 **Atom types and partial charges for BI-1001.**
(DOC)

Table S2 **Atom types and partial charges for CX14442.**
(DOC)

PDB S1 **The coordinates of the MD-simulated structure for BI-1001 in complex with HIV-1 IN CCD.**
(PDB)

PDB S2 **The coordinates of the MD-simulated structure for CX14442 in complex with HIV-1 IN CCD.**
(PDB)

PDB S3 **The coordinates of the MD-simulated structure for LEDGF/p75 in complex with HIV-1 IN CCD.**
(PDB)
