# Supplementary figures and images for: Molecular Modeling Study on the Allosteric Inhibition Mechanism of HIV-1 Integrase by LEDGF/p75 Binding Site Inhibitors
Source: PLoS One. 2014 Mar 5;9(3):e90799. doi: 10.1371/journal.pone.0090799 (PMC3944435; doi:10.1371/journal.pone.0090799)

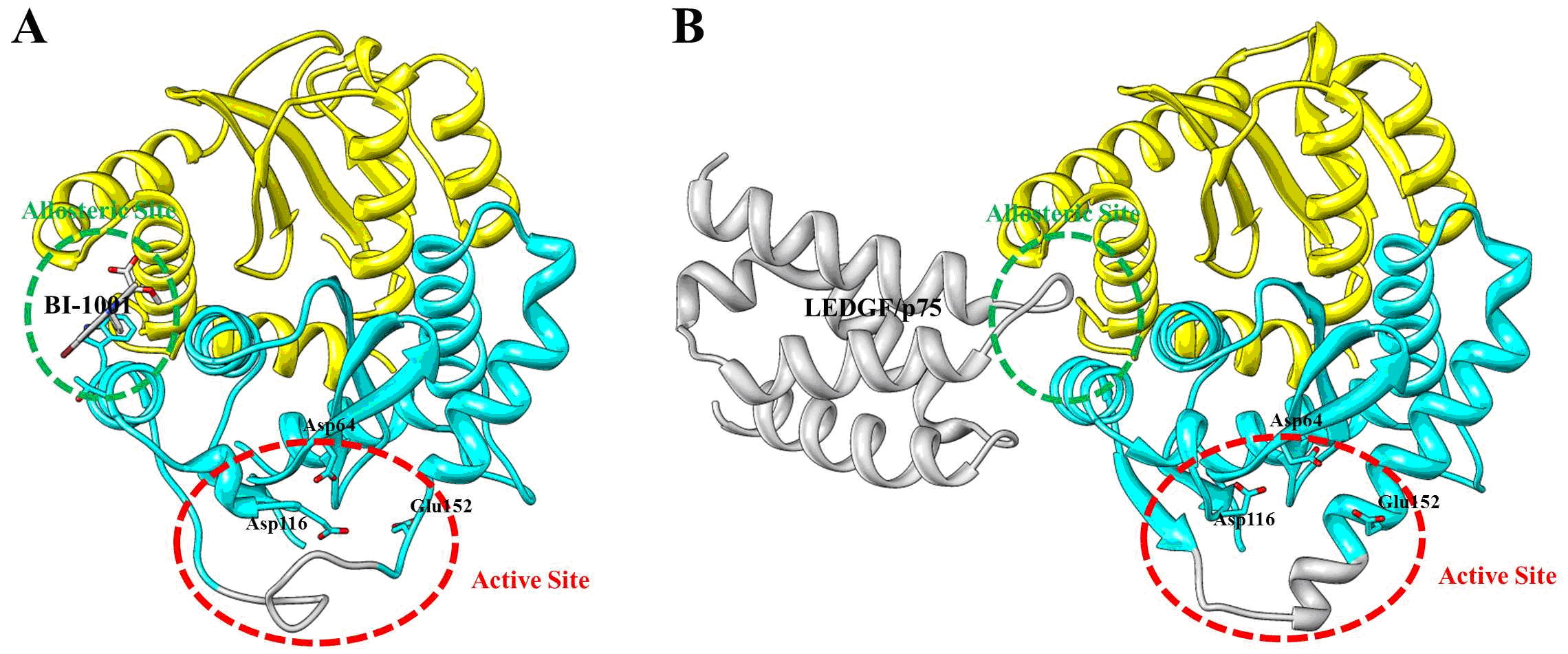

Supplement: Figure S1 — Structural models of BI-1001 and LEDGF/p75-bound HIV-1 IN CCD dimer complexes. (A) The modified crystal structures of BI-1001 in complex with HIV-1 IN CCD (PDB ID code 4DMN). (B) The crystal structures of LEDGF/p75 in complex with HIV-1 IN CCD (PDB ID code 2B4J). The protein is shown in the cartoon representation; the two monomers are colored yellow and cyan, respectively. The flexible 140 s loop (residues 140–149) is colored gray. HIV-1 IN active site residues (Asp64, Asp116, and Glu152) are shown in cyan stick. The LEDGINs and LEDGF/p75 are represented in gray stick and carton, respectively. (TIF) [file pone.0090799.s001.tif]
