# Supplementary material for: Molecular Modeling Study on the Allosteric Inhibition Mechanism of HIV-1 Integrase by LEDGF/p75 Binding Site Inhibitors
Source: PLoS One. 2014 Mar 5;9(3):e90799. doi: 10.1371/journal.pone.0090799 (PMC3944435; doi:10.1371/journal.pone.0090799)
Supplement: Table S1 — Atom types and partial charges for BI-1001. (DOC) [file pone.0090799.s002.doc]

**Table S1.** Atom types and partial charges for BI-1001


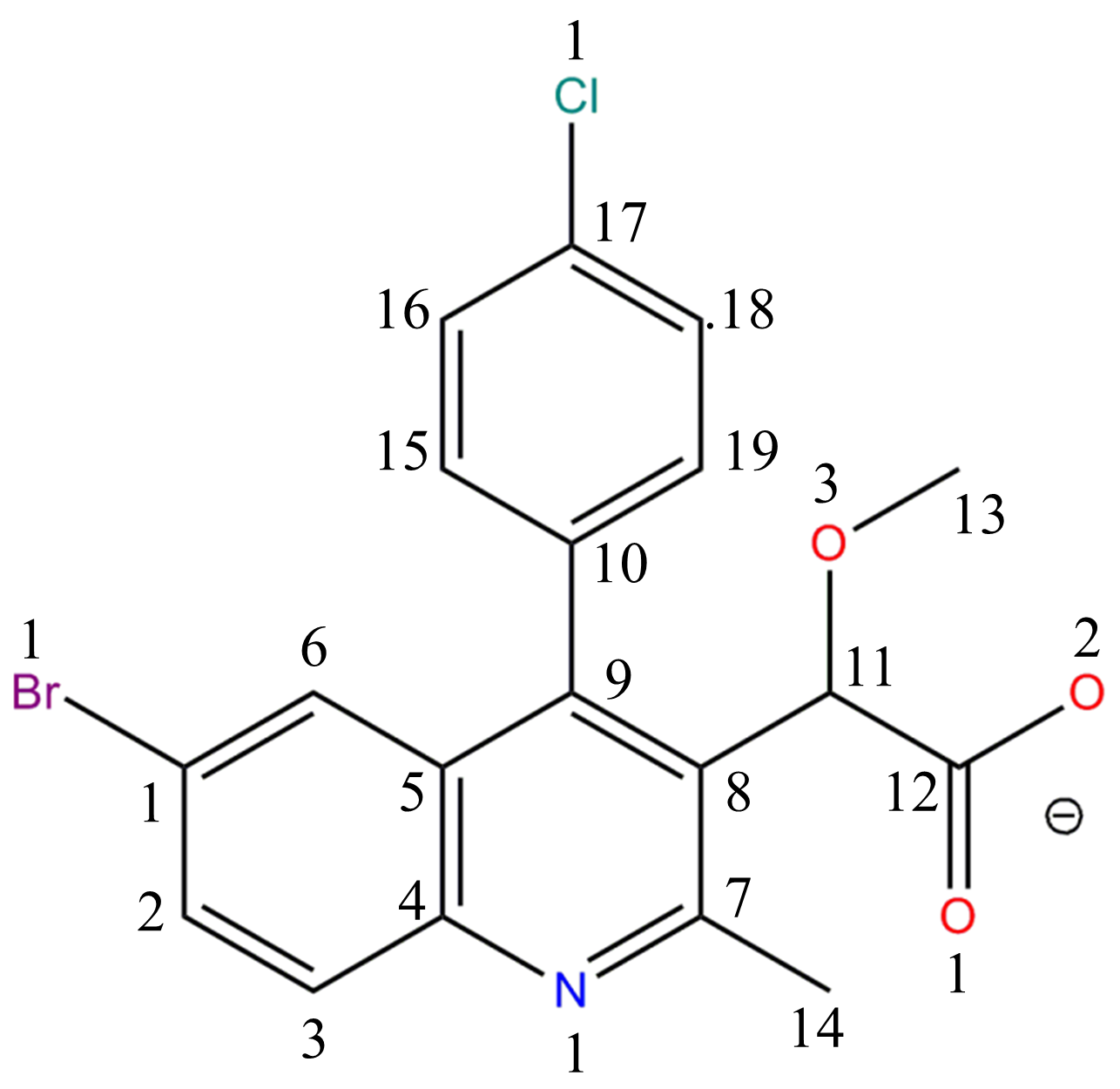


| Atom Name | Atom Type | Partial Charge |
| --- | --- | --- |
| Br1 | br | -0.1296 |
| C1 | ca | -0.2094 |
| C2 | ca | -0.0245 |
| C3 | ca | -0.4182 |
| C4 | ca | 0.7341 |
| C5 | ca | -0.2878 |
| C6 | ca | 0.1039 |
| C7 | ca | 1.0048 |
| C8 | ca | -0.8402 |
| C9 | cp | 0.1890 |
| C10 | cp | 0.2256 |
| C11 | c3 | 1.0931 |
| C12 | c | 0.5327 |
| C13 | c3 | 0.1940 |
| C14 | c3 | -0.7435 |
| C15 | ca | -0.1168 |
| C16 | ca | -0.1704 |
| C17 | ca | 0.0542 |
| C18 | ca | -0.1704 |
| C19 | ca | -0.1168 |
| Cl1 | cl | -0.1665 |
| H1 | ha | 0.1391 |
| H2 | ha | 0.2012 |
| H3 | ha | 0.0481 |
| H4 | h1 | -0.1397 |
| H5 | h1 | -0.0044 |
| H6 | h1 | -0.0044 |
| H7 | h1 | -0.0044 |
| H8 | hc | 0.2011 |
| H9 | hc | 0.2011 |
| H10 | hc | 0.2011 |
| H11 | ha | 0.1057 |
| H12 | ha | 0.1530 |

**Table S1.** Continued

| Atom Name | Atom Type | Partial Charge |
| --- | --- | --- |
| H13 | ha | 0.1530 |
| H14 | ha | 0.1057 |
| N1 | nb | -0.9305 |
| O1 | o | -0.7308 |
| O2 | o | -0.7308 |
| O3 | os | -0.7010 |

**Table S2.** Atom types and partial charges for CX14442


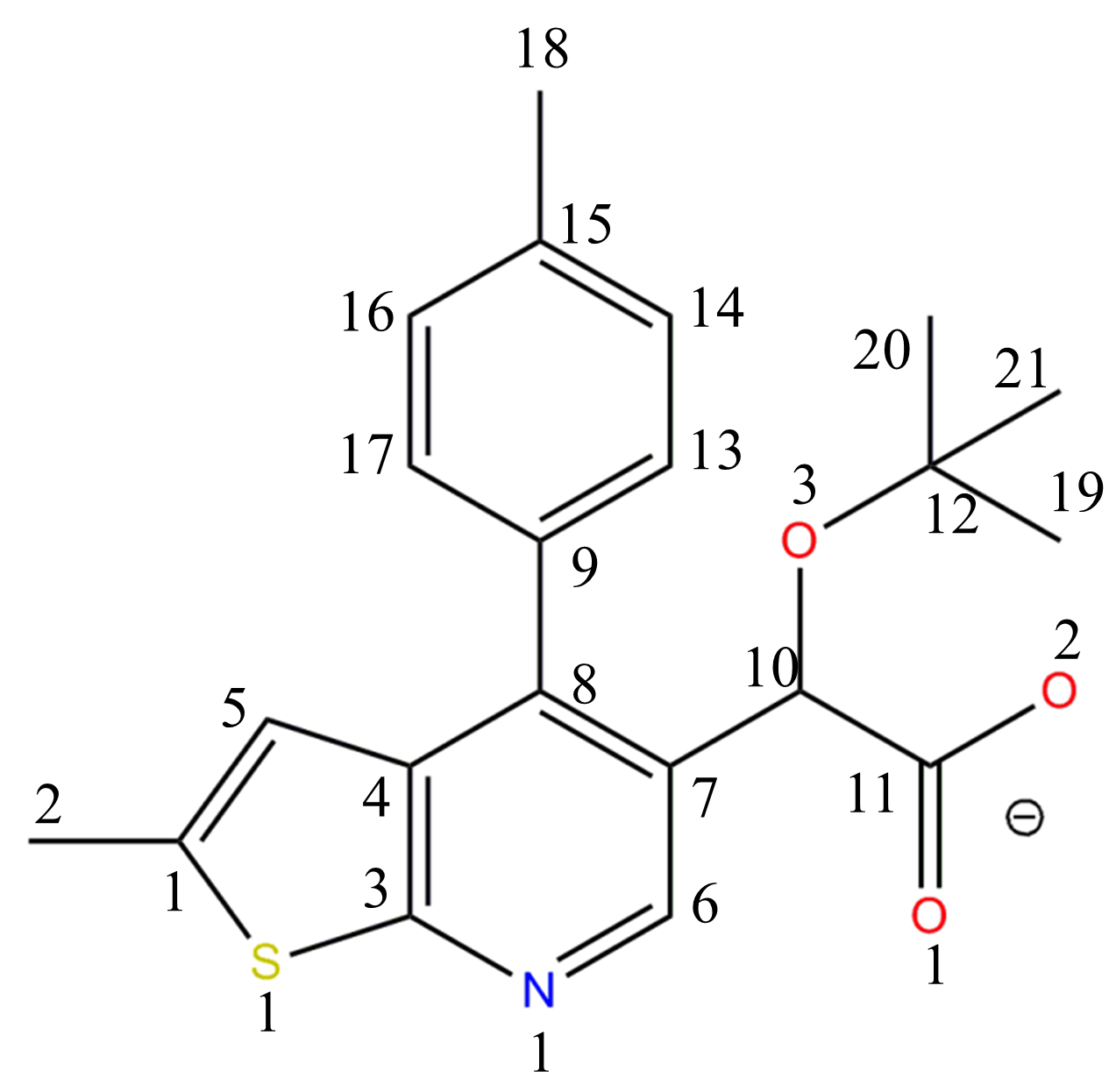


| Atom Name | Atom Type | Partial Charge |
| --- | --- | --- |
| N1 | nb | -0.6186 |
| C1 | cc | 0.1234 |
| O1 | o | -0.7861 |
| S1 | ss | -0.1966 |
| C2 | c3 | -0.2378 |
| O2 | o | -0.7861 |
| C3 | ca | 0.4315 |
| O3 | os | -0.9077 |
| C4 | ca | -0.0617 |
| C5 | cd | -0.3848 |
| C6 | ca | 0.3293 |
| C7 | ca | -0.2987 |
| C8 | cp | 0.0487 |
| C9 | cp | 0.0638 |
| C10 | c3 | 0.8214 |
| C11 | c | 0.6600 |
| C12 | c3 | 1.0948 |
| C13 | ca | 0.0106 |
| C14 | ca | -0.4826 |
| C15 | ca | 0.3911 |
| C16 | ca | -0.4826 |
| C17 | ca | 0.0106 |
| C18 | c3 | -0.5200 |
| C19 | c3 | -0.2380 |
| C20 | c3 | -0.2380 |
| C21 | c3 | -0.2380 |
| H1 | hc | 0.1370 |
| H2 | hc | 0.0871 |
| H3 | hc | 0.1370 |
| H4 | hc | 0.0871 |
| H5 | hc | 0.1370 |
| H6 | hc | 0.0871 |
| H7 | hc | 0.0016 |

**Table S2`.** Continued

| Atom Name | Atom Type | Partial Charge |
| --- | --- | --- |
| H8 | hc | 0.0016 |
| H9 | ha | 0.1877 |
| H10 | hc | 0.0016 |
| H11 | h4 | 0.1013 |
| H12 | hc | 0.0016 |
| H13 | hc | 0.0016 |
| H14 | hc | 0.0016 |
| H15 | hc | 0.0016 |
| H16 | hc | 0.0016 |
| H17 | hc | 0.0016 |
| H18 | h1 | -0.0875 |
| H19 | ha | 0.0975 |
| H20 | ha | 0.2044 |
| H21 | ha | 0.2044 |
| H22 | ha | 0.0975 |

**
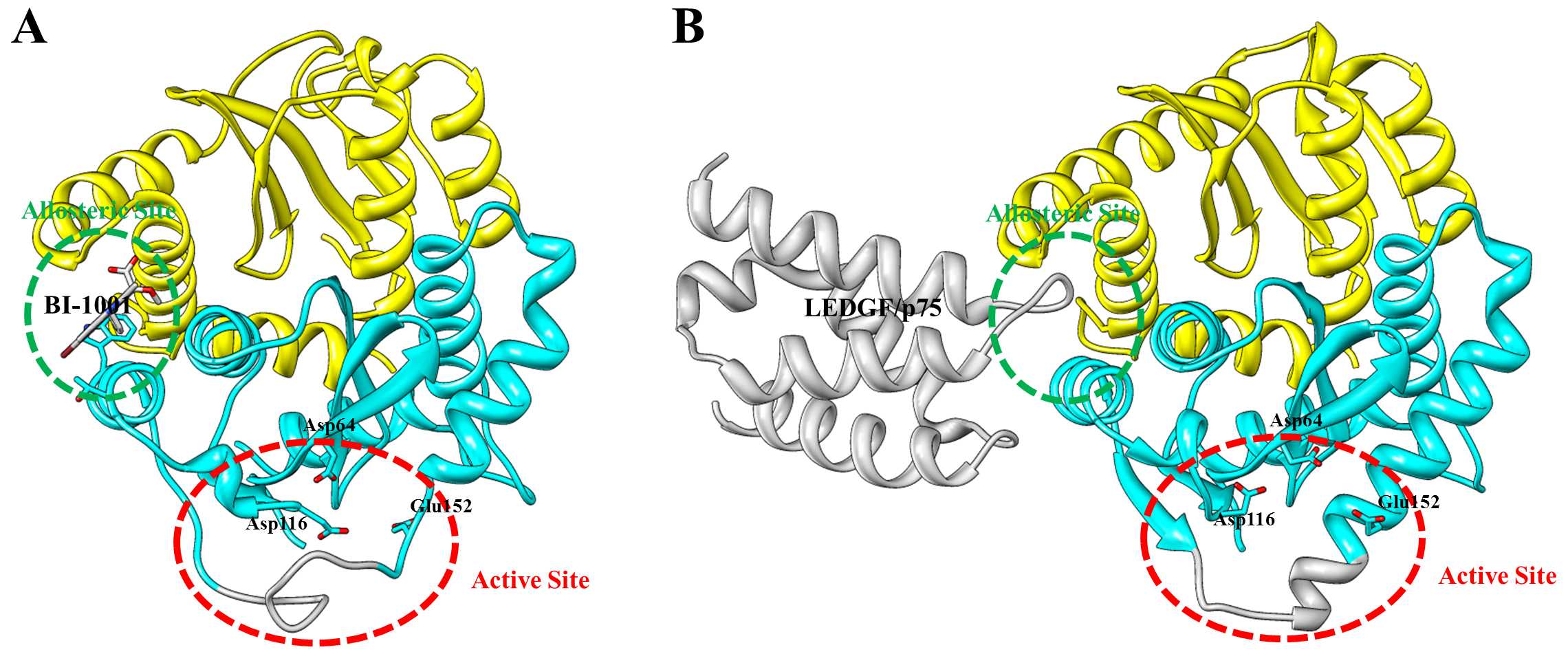
**

**Figure S1.** Structural models of BI-1001 and LEDGF/p75-bound HIV-1 IN CCD dimer complexes. (A) The modified crystal structures of BI-1001 in complex with HIV-1 IN CCD (PDB ID code 4DMN). (B) The crystal structures of LEDGF/p75 in complex with HIV-1 IN CCD (PDB ID code 2B4J). The protein is shown in the cartoon representation, the two monomers are colored yellow and cyan, respectively. The flexible 140s loop (residues 140-149) is colored gray. HIV-1 IN active site residues (Asp64, Asp116, and Glu152) are shown in cyan stick. The LEDGINs and LEDGF/p75 are represented in gray stick and carton, respectively.
