# Supplementary material for: Molecular Modeling Study on the Allosteric Inhibition Mechanism of HIV-1 Integrase by LEDGF/p75 Binding Site Inhibitors
Source: PLoS One. 2014 Mar 5;9(3):e90799. doi: 10.1371/journal.pone.0090799 (PMC3944435; doi:10.1371/journal.pone.0090799)
Supplement: Table S2 — Atom types and partial charges for CX14442. (DOC) [file pone.0090799.s003.doc]

**Table S2.** Atom types and partial charges for CX14442


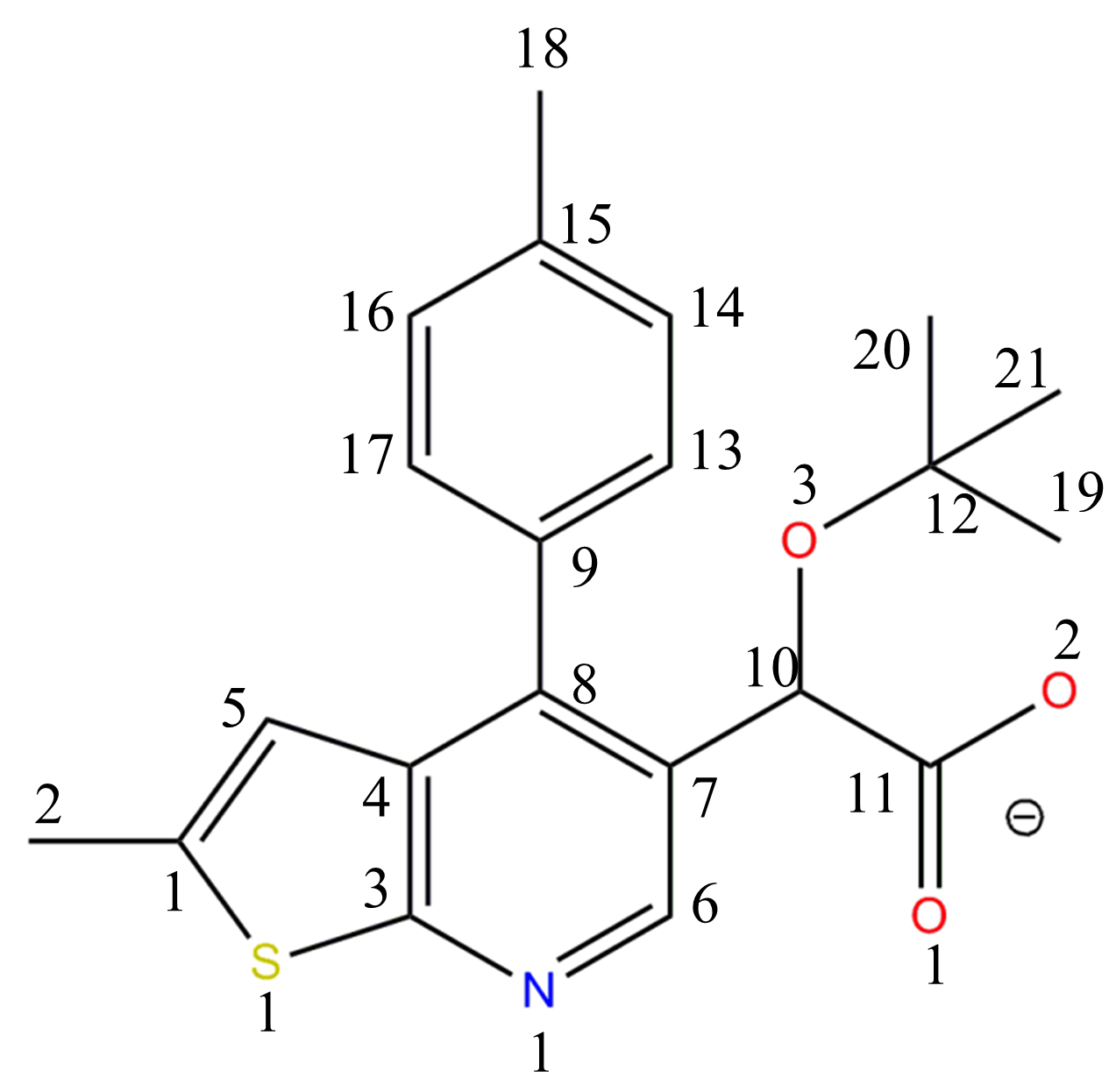


| Atom Name | Atom Type | Partial Charge |
| --- | --- | --- |
| N1 | nb | -0.6186 |
| C1 | cc | 0.1234 |
| O1 | o | -0.7861 |
| S1 | ss | -0.1966 |
| C2 | c3 | -0.2378 |
| O2 | o | -0.7861 |
| C3 | ca | 0.4315 |
| O3 | os | -0.9077 |
| C4 | ca | -0.0617 |
| C5 | cd | -0.3848 |
| C6 | ca | 0.3293 |
| C7 | ca | -0.2987 |
| C8 | cp | 0.0487 |
| C9 | cp | 0.0638 |
| C10 | c3 | 0.8214 |
| C11 | c | 0.6600 |
| C12 | c3 | 1.0948 |
| C13 | ca | 0.0106 |
| C14 | ca | -0.4826 |
| C15 | ca | 0.3911 |
| C16 | ca | -0.4826 |
| C17 | ca | 0.0106 |
| C18 | c3 | -0.5200 |
| C19 | c3 | -0.2380 |
| C20 | c3 | -0.2380 |
| C21 | c3 | -0.2380 |
| H1 | hc | 0.1370 |
| H2 | hc | 0.0871 |
| H3 | hc | 0.1370 |
| H4 | hc | 0.0871 |
| H5 | hc | 0.1370 |
| H6 | hc | 0.0871 |
| H7 | hc | 0.0016 |

**Table S2`.** Continued

| Atom Name | Atom Type | Partial Charge |
| --- | --- | --- |
| H8 | hc | 0.0016 |
| H9 | ha | 0.1877 |
| H10 | hc | 0.0016 |
| H11 | h4 | 0.1013 |
| H12 | hc | 0.0016 |
| H13 | hc | 0.0016 |
| H14 | hc | 0.0016 |
| H15 | hc | 0.0016 |
| H16 | hc | 0.0016 |
| H17 | hc | 0.0016 |
| H18 | h1 | -0.0875 |
| H19 | ha | 0.0975 |
| H20 | ha | 0.2044 |
| H21 | ha | 0.2044 |
| H22 | ha | 0.0975 |
